# High Cycle-life Shape Memory Polymer at High Temperature

**DOI:** 10.1038/srep33610

**Published:** 2016-09-19

**Authors:** Deyan Kong, Xinli Xiao

**Affiliations:** 1 MIIT Key Laboratory of Critical Materials Technology for New Energy Conversion and Storage, School of Chemistry and Chemical Engineering, Harbin Institute of Technology, No. 92 West Dazhi Street, Harbin 150001, PRC

## Abstract

High cycle-life is important for shape memory materials exposed to numerous cycles, and here we report shape memory polyimide that maintained both high shape fixity (*R*_*f*_) and shape recovery (*R*_*r*_) during the more than 1000 bending cycles tested. Its critical stress is 2.78 MPa at 250 °C, and the shape recovery process can produce stored energy of 0.218 J g^−1^ at the efficiency of 31.3%. Its high *R*_*f*_ is determined by the large difference in storage modulus at rubbery and glassy states, while the high *R*_*r*_ mainly originates from its permanent phase composed of strong π-π interactions and massive chain entanglements. Both difference in storage modulus and overall permanent phase were preserved during the bending deformation cycles, and thus high *R*_*f*_ and *R*_*r*_ were observed in every cycle and the high cycle-life will expand application areas of SMPs enormously.

Shape memory materials such as shape memory polymers (SMP) and shape memory alloys (SMA) have attracted more and more attentions from various fields, as they can be deformed into temporary shapes and then recover to the original shapes under suitable external stimuli^1^,^2^,^3^,^4^,^5^. Cycle-life represents the number of consecutive shape memory cycles that SMA or SMP can achieve without noticeable decrease in shape recovery (*R*_*r*_) and shape fixity (*R*_*f*_), and high cycle-life is crucial to applications demanding numerous shape memory cycles^2^,^6^,^7^,^8^,^9^,^10^. Bending deformation has been widely used to characterize the cycle-life of SMAs and SMPs, as large deflection can be achieved through small strains in bending^2^,^6^,^7^,^11^. SMAs exhibit high cycle-life as they can maintain both high *R*_*f*_ and *R*_*r*_ in thousands of or more bending deformation cycles before downgrade of shape memory performance or rupture of the sample in most cases^2^,^5^,^6^,^7^.

SMP possesses advantages such as easy process, large recoverable strains and light weight over SMA, but the high cycle-life SMP is rarely reported^12^,^13^,^14^,^15^,^16^. When SMP is used as bio-surgical implant, it is discarded after use and thus only 1 cycle is performed^17^,^18^. Most SMPs were exposed to 3 or 5 consecutive cycles, and the reported SMPs with more cycles usually showed decrease in shape memory performance after certain cycles^19^,^20^,^21^,^22^. For example, the commercially available SMP Veriflex^TM^ exhibited a steady decrease in *R*_*r*_ after 19 thermomechanical cycles^23^, the Si-O-Si cross-linked hybrid polyurethanes experienced lower *R*_*f*_ in water with the increase of cycles during the more than 50 bending tests^24^, and the polyether-based polyurethane manifested a loss in *R*_*r*_ by about 4% after 200 bending cycles^11^. As far as we know, there is no report about high cycle-life SMP that can maintain both high *R*_*f*_ and *R*_*r*_ after several hundred or more shape memory cycles at high temperature until now^25^,^26^,^27^,^28^,^29^,^30^,^31^.

SMPs can store strain and stress that correspond to certain energy, and the ability of shape memory materials to perform mechanical work against external loads during the shape recovery process is of great importance for practical applications^32^. The shape deformation energy of some common SMPs such as polyurethane and polystyrene have been reported, but the stored elastic energy during shape recovery process is seldom studied since most SMPs undergo stress-free shape recovery^33^,^34^. Tiller *et al*. have proposed a process to study the stretching energy and stored energy of shape memory natural rubber by numerical integration of force versus crosshead travel plot of a tensile tester during a shape-memory cycle^35^,^36^. However, there is no report about the energy-storage potential of high temperature SMPs so far.

In the current report, high temperature SMP with high cycle-life is obtained from the polyimide possessing large content of aromatic groups and highly twisted molecular chains. The stretching energy and stored energy of the polyimide during a shape memory cycle, as well as its critical stress at 250 °C are studied. The huge difference in storage modulus at rubbery and glassy states leads to high *R*_*f*_, while the strong π-π interactions and massive chain entanglements caused by its particular structure act as permanent phase and produce high *R*_*r*_. The polyimide maintained both high *R*_*f*_ and *R*_*r*_ of about 100% during the more than 1000 shape memory cycles tested, and the possible mechanisms of its high cycle-life are discussed. The high cycle-life shape memory polyimide has offered suitable candidate for high temperature applications requiring numerous cycles such as reversible actuators and deployable hinges.

## Results

### Molecular weight and structure

**Figure 1:**
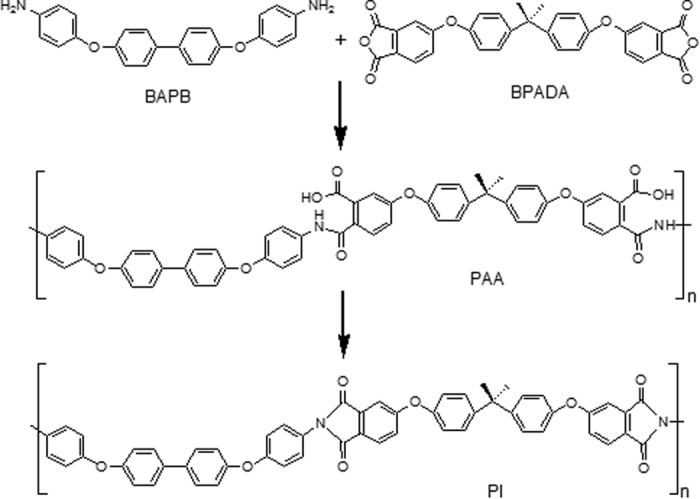
Two-step polycondensation process of the high cycle-life shape memory polyimide.

The high cycle-life shape memory polyimide was synthesized with 4,4′-(1,1′-biphenyl-4,4′-diyldioxy)-dianiline (BAPB) and bis phenol A dianhydride (BPADA), and the two-step polycondensation process is manifested in Fig. 1. Its number average molecular weight (*M*_*n*_) is 24.6 kg/mol, and the polydispersity index (PDI) is 1.53.

Structure of the polyimide was characterized with infrared (IR), and the IR spectra are shown in the supplementary information (Figure S1). The characteristic IR peaks of polyimide such as asymmetric stretching of C = O at 1779 cm^−1^, symmetric stretching of C = O at 1722 cm^−1^ and stretching vibration of C-N-C at 1374 cm^−1^ indicate the formation of polyimide. The absent IR peaks of carbonyl of isoimide at 1795–1820 or 921–934 cm^−1^ manifest the lack of isoimides. These results indicate that the high cycle-life shape memory polyimide was fully imidized^37^.

### Thermomechanical properties and thermal stability

**Figure 2:**
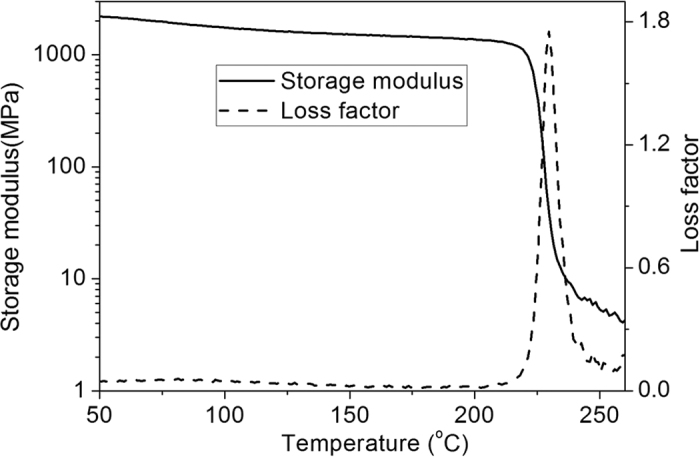
Thermomechanical properties of high cycle-life shape memory polyimide. The solid line and dashed line represent its storage modulus and loss factor, respectively.

Dynamic mechanical analysis (DMA) was employed to characterize thermomechanical properties of the high cycle-life shape memory polyimide, and the evolutions of its loss factor (tan δ) and tensile storage modulus versus temperature are shown in Fig. 2. The peak of tan δ is employed as its glass transition temperature (*T*_*g*_), and the *T*_*g*_ of 229.6 °C indicates that it is suitable for high temperature applications. The storage modulus is rather high and decreases slowly with the increase of temperature at glassy state, and the values at 60 °C and 199.6 °C (*T*_*g*_-30 °C) are 2.12 GPa and 1.37 GPa, respectively. Then the storage modulus undergoes a huge drop during the glass transition process and becomes rather low at rubbery state, and its value at 249.6 °C (*T*_*g*_ + 20 °C) is 6.5 MPa.

**Figure 3:**
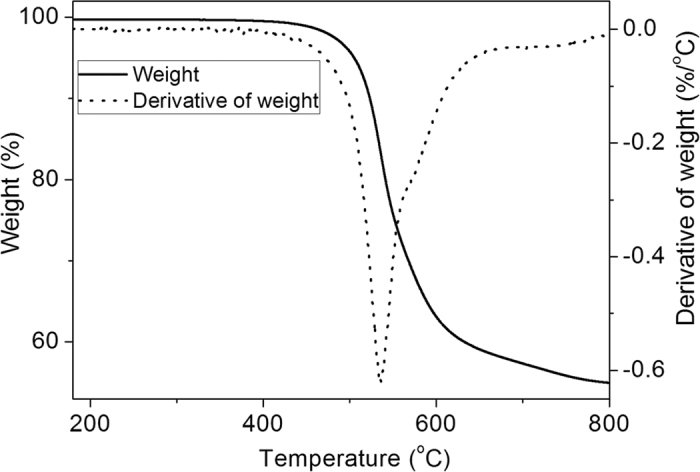
TGA spectra of high cycle-life shape memory polyimide. The solid line and dotted line represent the weight and derivative of weight versus temperature, respectively.

Thermal stability of the high cycle-life shape memory polyimide was characterized with thermal gravimetric analysis (TGA), and the TGA results are shown in Fig. 3. It is observed that the effective decomposition temperature (*T*_*d*_, 5% weight loss) is 505 °C, and the major pyrogenic decomposition started at 536 °C. These results indicate that this polyimide is highly thermally stable.

### Shape recovery temperature and the critical stress

*R*_*f*_ and *R*_*r*_ are two most important factors in evaluating shape memory performances, as they represent the capability of the shape memory material to fix temporary shape and return to initial shape, respectively^2^. *R*_*f*_ of bending deformation can be calculated with Equation 1:


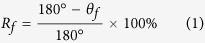


Here *θ*_*f*_ represents the released angle after cooling and it is measured between the two straight ends of the bended sample. *R*_*r*_ can be calculated with Equation 2:


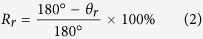


The *θ*_*r*_ represents the recovered angle and the schematic illustration of a shape memory bending cycle is shown in the supplementary information (Figure S2).

The constitutive models have proposed that deformation conditions such as temperature and stress can affect the shape memory behavior of SMPs^13^,^38^. The influence of shape recovery temperature on *R*_*r*_ of the polyimide was studied, and its recovered shapes at *T*_*g*_ + 20 °C, *T*_*g*_ + 30 °C, *T*_*g*_ + 40 °C, *T*_*g*_ + 50 °C, *T*_*g*_ + 60 °C and *T*_*g*_ + 70 °C are manifested in the supporting information (Figure S3). It is observed that the polyimide can recover to its original shape completely at all these temperatures, i.e. its *R*_*r*_ is 100% in all cases. These results indicate that this polyimide possesses wide application temperature ranges, and *T*_*g*_ + 20 °C is employed as its shape recovery temperature for convenience.

Elastic deformation can be recovered while plastic deformation is unrecoverable, and therefore plastic deformation should be avoided in SMPs^2^,^10^. When the bended polyimide was positioned upside-down in the oven at *T*_*g*_ + 20 °C (250 °C), it was not prolonging plastically under its gravitation. Compared with the *R*_*r*_ of 100% in normally positioned polyimide, *R*_*r*_ was 63% when the polyimide was positioned upside-down in the oven, as shown in the supporting information (Figure S4). These results indicate that gravitation contributes partially to the shape recovery of the polyimide when it is positioned normally, and its shape recovery process can produce energy to lift itself against gravitation.

**Figure 4:**
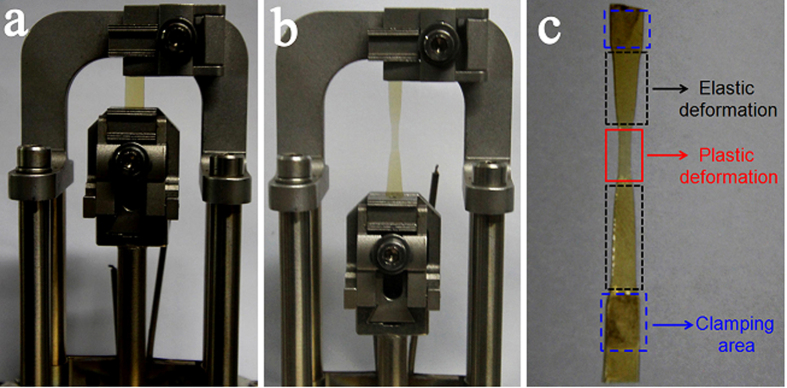
Critical stress for plastic deformation of the shape memory polyimide at Tg + 20 °C. (**a**) Initial sample fastened by DMA clamp, (**b**) the sample stretched at 250 °C with critical stress, and (**c**) indication of elastic deformation and plastic deformation.

The critical stress for plastic deformation of SMAs have been studied extensively^39^, but there is no report about critical stress of SMPs until now. Here critical stress of the polyimide was examined and the initial flat lath was fastened with DMA clamps, as shown in Fig. 4a. Then it was stretched at 250 °C and when the load increased to critical stress, plastic deformation appeared (Fig. 4b). There is a distinct difference between elastic deformation and plastic deformation of the shape memory polyimide, as illustrated in Fig. 4c.

**Figure 5:**
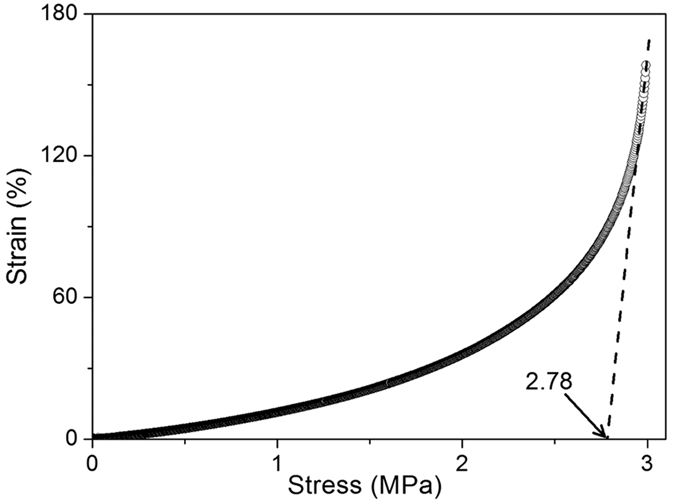
Evolution of strain versus stress of the shape memory polyimide at 250 °C.

The value of critical stress for plastic deformation of the polyimide at shape recovery temperature (*T*_*g*_ + 20 °C) is calculated from the stress-strain curve (Fig. 5). At early stage the increment of stress led to a gradual increase of strain, but the critical stress resulted in a sharp increase in strain. The critical value obtained from tangent of the stress-strain curve is 2.78 MPa.

### Energy-storage capacity and efficiency

**Figure 6:**
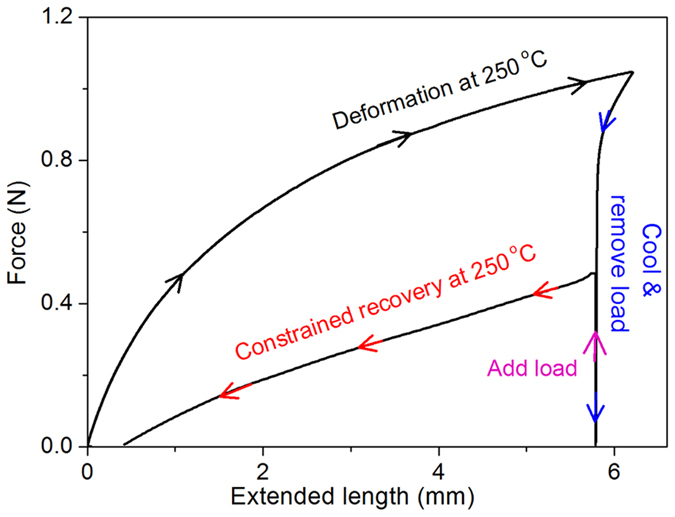
Stretching and stored energy of the polyimide in a shape memory cycle at 250 °C.

The mechanical work that SMP can perform during a shape memory process is closely related with its energy-storage potentials^2^. The energy-storage capacity of some low to medium temperature (20–100 °C) SMPs such as natural rubber and polystyrene have been studied^32^,^33^,^34^,^35^,^36^, but the reported methods for samples of 1 mm or more in thickness are not suitable for the shape memory polyimide due to its low thickness of about 0.1 mm. Therefore, the energy-storage potentials of the polyimide was characterized with DMA. The polyimide was fastened with two clamps and then stretched at 250 °C, which corresponds to the “Deformation at 250 °C” in Fig. 6. The stretching energy (*W*_*stret*_) to stretch the polyimide was calculated by numerical integration of the load versus clamp travel curves with Equation 3.


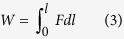


Then the temperature was decreased to 170° and the load was removed, and the temporary shape was fixed. A load was applied to the polyimide and the temperature was increased to 250 °C again to promote the shape recovery, corresponding to the “Constrained recovery at 250 °C” in Fig. 6. The elastically stored energy (*W*_*store*_) during the shape recovery is also equal to Equation 3 of the recovery path. The calculated stretching energy and stored energy of the polyimide at 250 °C are 0.697 J g^−1^ and 0.218 J g^−1^, respectively. The efficiency *η* is the ratio of stored to stretching energy, and the polyimide possesses *η* of 31.3%.

### High cycle-life shape memory properties

**Figure 7:**
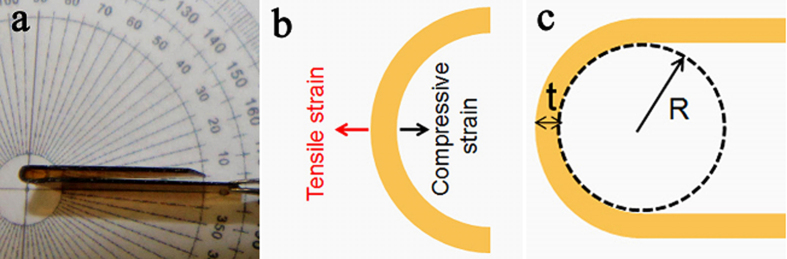
Bending deformation of shape memory polyimide. (**a**) Image of a bended polyimide, (**b**) illustration of compressive strain and tensile strain of inner and outer surfaces, and (**c**) mechanical model of bending deformation.

Similar to the common characterization method of cycle-life^2^,^4^,^5^,^6^,^7^,^11^,^40^, bending deformation was also employed to characterize cycle-life of this shape memory polyimide and the image of a bended sample is shown in Fig. 7a. The inner surface and outer surface of the bend undergo compressive strain and tensile strain respectively, as manifested in Fig. 7b. The inner and outer surface strains are determined by film thickness (*t*) and radius of the curvature (*R*)^41^, and the mechanical model of bending deformation is shown in Fig. 7c.

The compressive strain of inner surface (*ε*_*inner*_) is calculated with Equation 4.


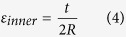


The tensile strain of outer surface (*ε*_*outer*_) is calculated with Equation 5.


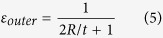


For the bended shape memory polyimide with film thickness of 0.12 mm and curvature radius of 0.15 mm, the compressive strain of inner surface and tensile strain of outer surface are 0.400 and 0.286, respectively.

**Figure 8:**
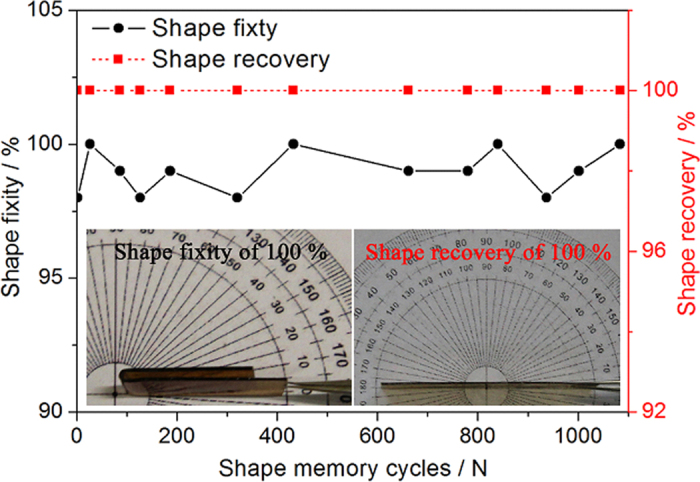
Overview of shape memory performances of the polyimide during the bending cycles. The inlets are images of complete shape fixity and shape recovery states.

The high cycle-life shape memory polyimide showed excellent shape memory performances, and *R*_*f*_ varied from 98% to 100% while *R*_*r*_ was always 100% during the more than 1000 cycles tested. Overview of shape memory performances of the polyimide in these cycles is manifested in Fig. 8, and the images of complete shape fixity and shape recovery states are shown as the inlets.

**Figure 9:**
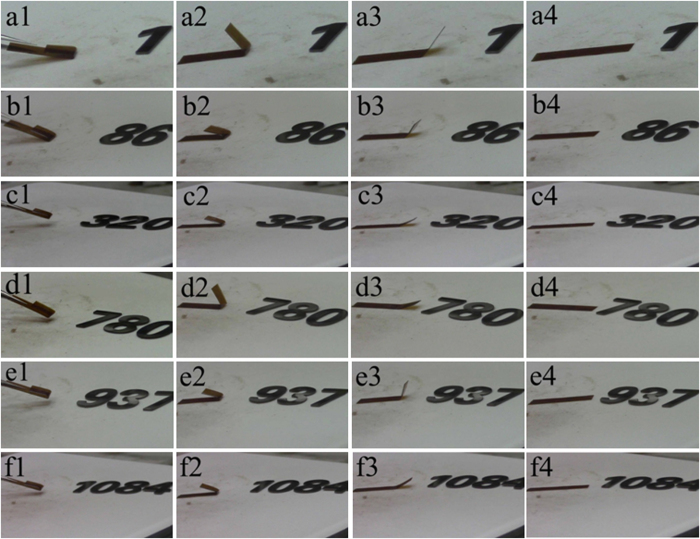
Shape recovery processes of the high cycle-life shape memory polyimide. (**a1**) 1st recovery at 0 s, (**a2**) 4 s, (**a3**) 7 s and (**a4**) 10 s; (**b1**) 86th recovery at 0 s, (**b2**) 1 s, (**b3**) 5 s and (**b4**) 11 s; (**c1**) 320th recovery at 0 s, (**c2**) 2 s, (**c3**) 7 s and (**c4**) 11 s; (**d1**) 780th recovery at 0 s, (**d2**) 7 s, (**d3**) 10 s and (**d4**) 15 s; (**e1**) 937th recovery at 0 s, (**e2**) 2 s, (**e3**) 5 s and (**e4**) 10 s; (**f1**) 1084th recovery at 0 s, (**f2**) 3 s, (**f3**) 5 s and (**f4**) 11 s.

Detailed shape recovery processes of the 1st, 86th, 320th, 780th, 937th and 1084th cycles of the high cycle-life shape memory polyimide are manifested in Fig. 9, and the movies showing these processes are illustrated in supplementary information as Movie S1, Movie S2, Movie S3, Movie S4, Movie S5 and Movie S6, respectively.

**Figure 10:**
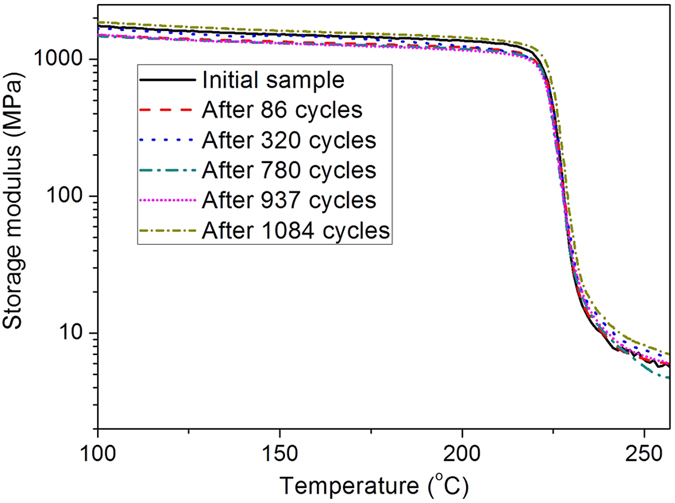
Storage modulus of the high cycle-life shape memory polyimide after different bending deformation cycles.

It has been observed that the high *R*_*f*_ of SMP is closely related with the large difference in its storage modulus before and after the shape transition temperature^2^,^12^,^20^,^28^. Here the storage modulus of the high cycle-life shape memory polyimide after different bending deformation cycles were characterized, as illustrated in Fig. 10. It is clearly manifested that the sample showed similar storage modulus after different shape memory cycles, and the huge difference in their values at glassy and rubbery states was preserved. The ratios of storage modulus at glassy state (*T*_*g*_-30 °C) to that at rubbery state (*T*_*g*_ + 20 °C) for the initial sample and those after 86, 320, 780, 937 and 1084 shape memory cycles are 211, 189, 165, 206, 172 and 176, respectively.

**Figure 11:**
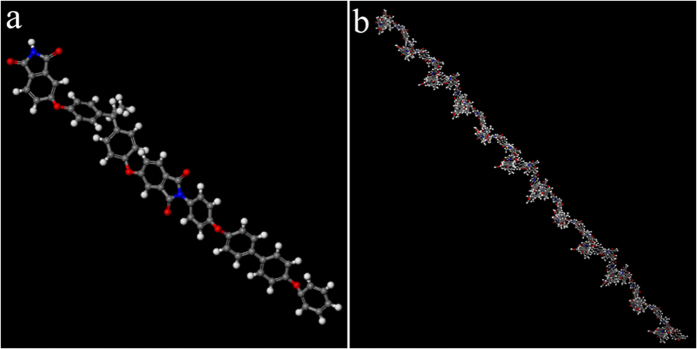
3D illustration of molecular structures of high cycle-life shape memory polyimide. (**a**) Its repeating unit, and (**b**) its twisted molecular chain. C, H, O and N atoms are manifested with grey, white, red and blue balls, respectively.

It is generally accepted that *R*_*r*_ of SMP is mainly determined by the physical or chemical crosslinkers that act as permanent phase^2^,^12^,^20^. For some common thermoplastic SMPs, the permanent phase is mainly composed of chain entanglements^2^. For thermoplastic shape memory polyimide, the permanent phase is composed of chain entanglements and π-π interactions^12^,^20^,^26^. As for the high cycle-life shape memory polyimide, *R*_*r*_is associated with the permanent phase caused by its special molecular structures. As shown in three-dimensional (3D) illustration of its repeating unit (Fig. 11a), this polyimide contains large content of aromatic groups, which will produce strong intermolecular π-π interactions. Moreover, such a polyimide molecular chain with *M*_*n*_ of 24.6 kg/mol is composed of 27 repeating units, and the chain is highly twisted like a spring (Fig. 11b). Such highly twisted chains are apt to crosslink with each other physically and form massive chain entanglements. As a result, the permanent phase composed of strong π-π interactions and massive chain entanglements will lead to high *R*_*r*_ of the polyimide.

**Figure 12:**
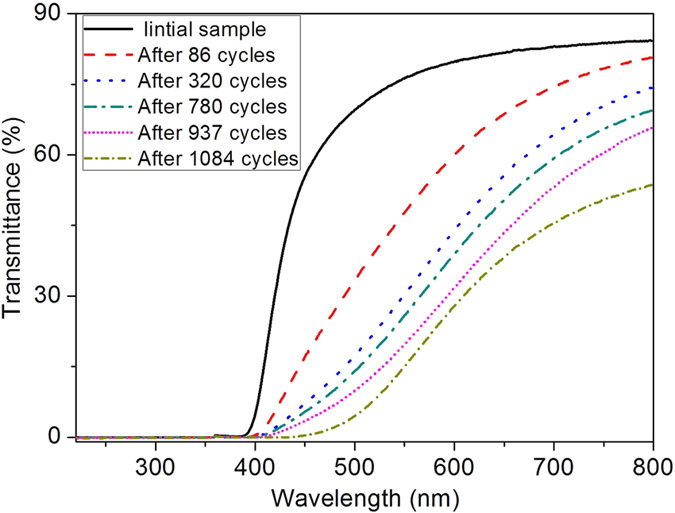
UV-Vis transmittance of high cycle-life shape memory polyimide after different bending deformation cycles.

The decrease in *R*_*r*_ of some SMPs after certain bending deformation cycles have demonstrated that some negative factors such as ageing process during successive shape memory cycles will cause damages to the permanent phase^2^,^11^. *R*_*r*_ of the high cycle-life shape memory polyimide was always 100% from the 1st to the 1084th shape memory cycles examined, indicating that its overall permanent phase was not deteriorated although chain entanglements will be inevitably damaged during the bending deformation cycles like common SMPs. UV-Vis transmittance of the polyimide after different cycles are shown in Fig. 12, and the decrease in transmittance manifests that the intermolecular π-π interactions get stronger with the increase of cycle numbers.

## Discussions

In the current report, high cycle-life SMP that exhibited *R*_*f*_ of 98-100% and *R*_*r*_ of 100% during the more than 1000 bending deformation cycles at 250 °C was obtained from shape memory BPADA/BAPB polyimide. *R*_*r*_ of 100% is obtained at shape recovery temperatures from 250 °C to 300 °C, indicating that this polyimide possesses rather wide application temperature ranges. The force produced in the shape recovery process can work against gravitation, and the critical stress to stretch the polyimide without plastic deformation at 250 °C is 2.78 MPa. The stretching energy and stored energy in a shape memory cycle at 250 °C are 0.697 J g^−1^ and 0.218 J g^−1^, and the energy efficiency of the polyimide is 31.3%. The mechanical work that the polyimide can perform is of great importance for its practical applications.

High *R*_*f*_ of the polyimide is mainly caused by the huge difference in storage modulus at rubbery and glassy states, as the low modulus favors deformation of initial shape at high temperature while the high modulus benefits fixing of temporary shape at low temperature^2^,^4^,^11^,^20^,^25^. The large difference in storage modulus at glassy and rubbery states was preserved during the shape memory cycles, as the value at *T*_*g*_ −30 °C was more than one hundred times higher than that at *T*_*g*_ + 20 °C in any case. Therefore, high *R*_*f*_ was maintained during the more than one thousand bending deformation cycles.

According to the shape memory mechanism of SMPs, *R*_*r*_ is closely related with its permanent phase and damages to the permanent phase will lead to lower *R*_*r*_. The BPADA/BAPB polyimide possesses large content of aromatic groups and highly twisted molecular chains, which produce strong π-π interactions and massive chain entanglements that act as permanent phase. The repeated thermomechanical shape memory cycles will cause damages to the chain entanglements, and *R*_*r*_ of common SMPs such as polyurethane decreased after certain cycles. However, π-π interactions of the polyimide got stronger with more thermomechanical cycles and the increased π-π interactions can offset the damages to chain entanglements^2^,^12^. As a result, overall permanent phase of the polyimide was not deteriorated and thus complete shape recovery was maintained during the bending deformation cycles.

In summary, shape recovery of this shape memory polyimide can produce energy of 0.218 J g^−1^ at 250 °C. The preservation of large difference in storage modulus at different states and maintenance of overall permanent phase endow this polyimide with high cycle-life. The high cycle-life shape memory polyimide can satisfy applications demanding numerous cycles at high temperature such as reversible actuators.

## Methods

### Materials

BAPB (98%) and BPADA (98%) were purchased from TCI and used without further purification. Dimethylformamide (DMF) was bought from Sinopharm Group Co. Ltd and dried with CaH_2_ before use.

### Synthesis of high cycle-life shape memory polyimide

5 m mol BAPB was added into the three-necked flask containing 40 ml DMF and then stirred under nitrogen until it was dissolved completely. 5 m mol BPADA was added into the BAPB solution and then stirred 24 hours under nitrogen at room temperature to produce poly(amic acid) (PAA). After elimination of bubbles in vacuum chamber, the PAA was transferred onto a clear glass sheet and underwent step-wise curing at 70, 100, 150, 180, 220 and 260 °C for 2 h, respectively. The polyimide was removed from substrate in water and then dried at 130 °C.

### Molecular weight and structure characterization

Molecular weight of the polyimide was characterized by size exculsion chromotography with Waters 2414 at 35 °C, and DMF was employed as the eluent. Its structure was characterized by Infrared (IR) with Thermo Nicolet Nexus 870 from 600 to 4000 cm^−1^ at the interval of 1 cm^−1^.

### UV-Vis characterization

Ultraviolet-visible (UV-Vis) transmittance was characterized with Persee T6 Ultraviolet-visible spectrophotometer from 200 to 800 nm at the interval of 1 nm.

### Thermomechanical property and thermal stability characterization

Thermomechanical property of the polyimide was determined with TA instrument DMA Q800 in tensile mode at the frequency of 1 Hz, and the heating rate was 4 °C/min. Its thermal stability was characterized with TA instrument Q500 TGA at the heating rate of 10 °C/min under nitrogen.

### Recovery temperature effect characterization

A typical shape memory process of the polyimide was carried out as follows: ① the flat polyimide lath was bended at *T*_*g*_ + 20 °C; ② it was cooled to room temperature with the deformation stress and the temporary shape was fixed; ③ the temporary shape was heated to *T*_*g*_ + 20 °C and it recovered to its original shape. The effect of shape recovery temperature on *R*_*r*_ was analyzed by studying the recovery at *T*_*g*_ + 20 °C, *T*_*g*_ + 30 °C, *T*_*g*_ + 40 °C, *T*_*g*_ + 50 °C, *T*_*g*_ + 60 °C and *T*_*g*_ + 70 °C, respectively.

### Shape recovery against gravitation characterization

A stainless-steel sheet was placed inside the angle between two ends of the bended polyimide lath, and then they were positioned onto the holding frame inside the oven. The shape recovery was programmed at *T*_*g*_ + 20 °C and the polyimide beneath the stainless-steel sheet recovered against gravitation.

### Critical stress characterization

Critical stress of the polyimide was characterized by stretching the sample fasten between two DMA clamps at 250 °C with an increasing load. At early stage the stress led to larger strains gradually, while the critical stress resulted in sharp increase of strain and irreversible plastic deformation.

### Stretching and stored energy characterization

A thermomechanical shape memory cycle was performed with DMA Q800 to determine the stretching energy and stored energy of the polyimide by following procedures: ① Ramp at 10 °C/min to 250 °C, ② Equilibrate at 250 °C and isothermal for 5 min, ③ Ramp force at 0.2 N/min to 1.05 N. ④ Ramp at 10 °C/min to 170 °C, ⑤ Ramp force at 0.2 N/min to 0.01 N, and isothermal for 2 min, ⑥ Ramp force to 0.48 N at 0.2 N/min ⑦ Ramp to 250 °C at 10 °C/min, ⑧ Ramp force to 0.01 N at 0.2 N/min.

### High cycle-life characterization

The bending deformation shape memory cycles at *T*_*g*_ + 20 °C was repeated more than 1000 cycles, and the same position of the sample was bended every time.

## Additional Information

**How to cite this article**: Kong, D. and Xiao, X. High Cycle-life Shape Memory Polymer at High Temperature. *Sci. Rep.* **6**, 33610; doi: 10.1038/srep33610 (2016).

## Supplementary Material

Supplementary Information

Supplementary Video 1

Supplementary Video 2

Supplementary Video 3

Supplementary Video 4

Supplementary Video 5

Supplementary Video 6
