# Supplementary material for: High Cycle-life Shape Memory Polymer at High Temperature
Source: Sci Rep. 2016 Sep 19;6:33610. doi: 10.1038/srep33610 (PMC5027533; doi:10.1038/srep33610)
Supplement: Supplementary Information [file srep33610-s1.doc]

Supplementary Information

**High cycle-life shape memory polymer at high temperature**

Deyan Kong, Xinli Xiao*

MIIT Key Laboratory of Critical Materials Technology for New Energy Conversion and Storage, School of Chemistry and Chemical Engineering, Harbin Institute of Technology, No.92 West Dazhi Street, Harbin 150001, PRC.

Corresponding author: Xinli Xiao, email: xiaoxinli@hit.edu.cn.

**Figure Captions**

Supplementary Figure S1. IR spectra of the high cycle-life shape memory polyimide.

Supplementary Figure S2. Schematic illustration of the shape fixity and shape recovery test in bending deformation.

Supplementary Figure S3. Complete shape recovery of the high cycle-life shape memory polyimide at recovery temperatures from *T_g_*+20 °C to *T_g_*+70 °C. (a) at *T_g_*+20 °C, (b) at *T_g_*+30 °C, (c) at *T_g_*+40 °C, (d) at *T_g_*+50 °C, (e) at *T_g_*+60 °C and (f) at *T_g_*+70 °C.

Supplementary Figure S4. Shape recovery of the polyimide against gravitation. (a) initial flat lath, (b) bended temporary shape, (c) stainless-steel sheet placed inside the bended polyimide, (d) shape recovery against gravitation at *T_g_*, (e) shape recovery against gravitation at *T_g_*+20 °C and (f) illustration of recovery angle at *T_g_*+20 °C against gravitation.


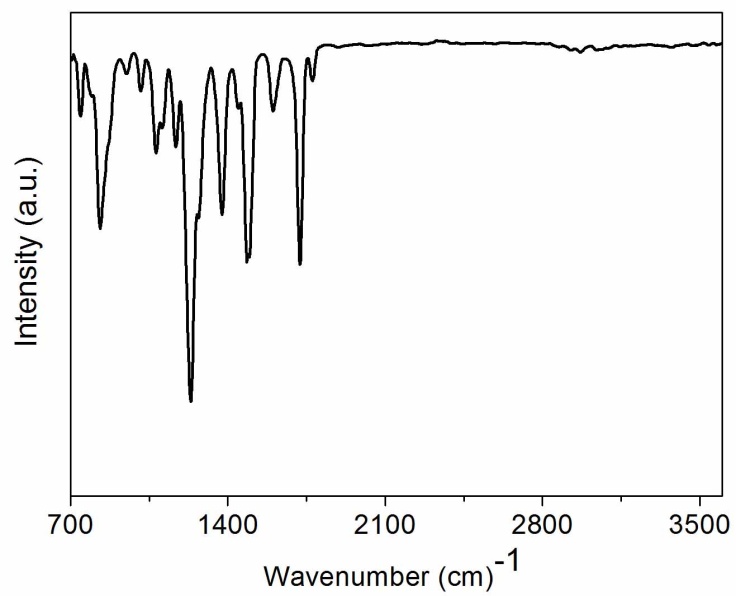


**Figure S1.** **IR spectra of the high cycle-life shape memory polyimide.**


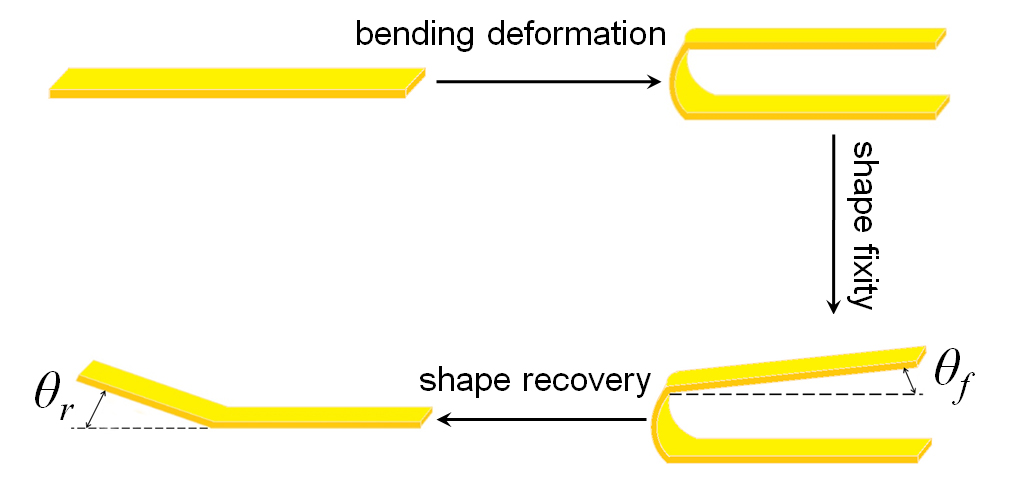


**Figure S2. Schematic illustration of the shape fixity and shape recovery test in bending deformation.**


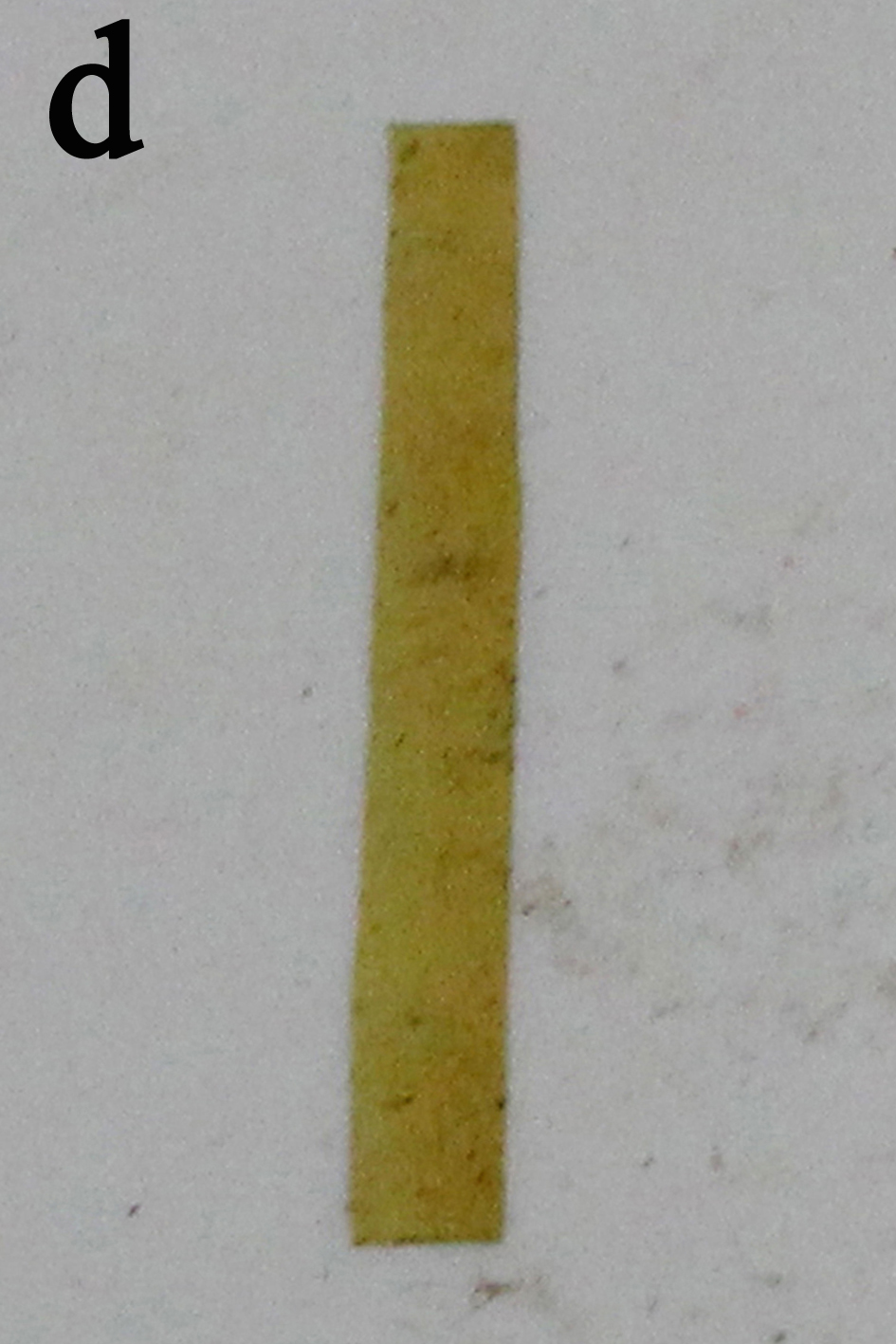

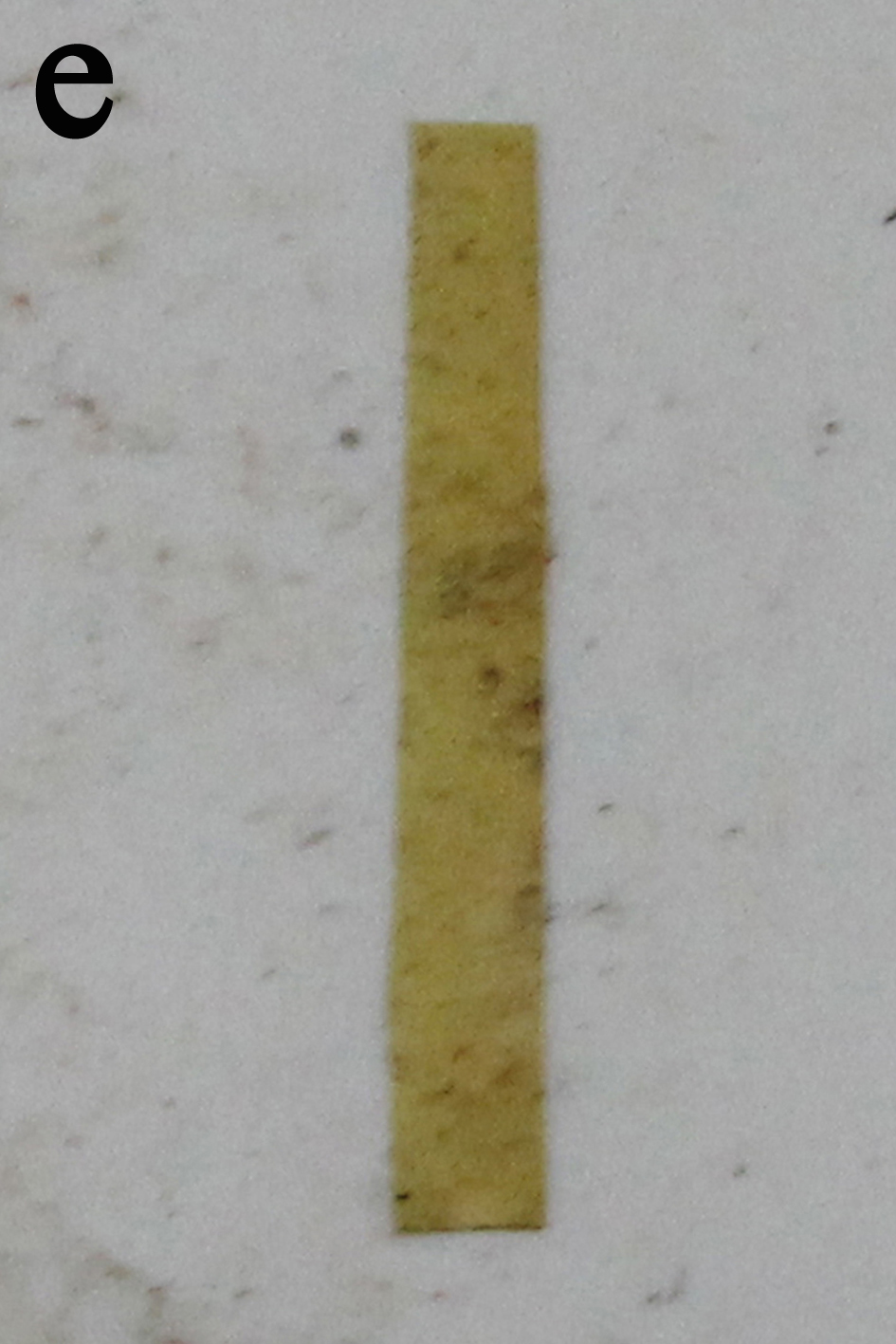

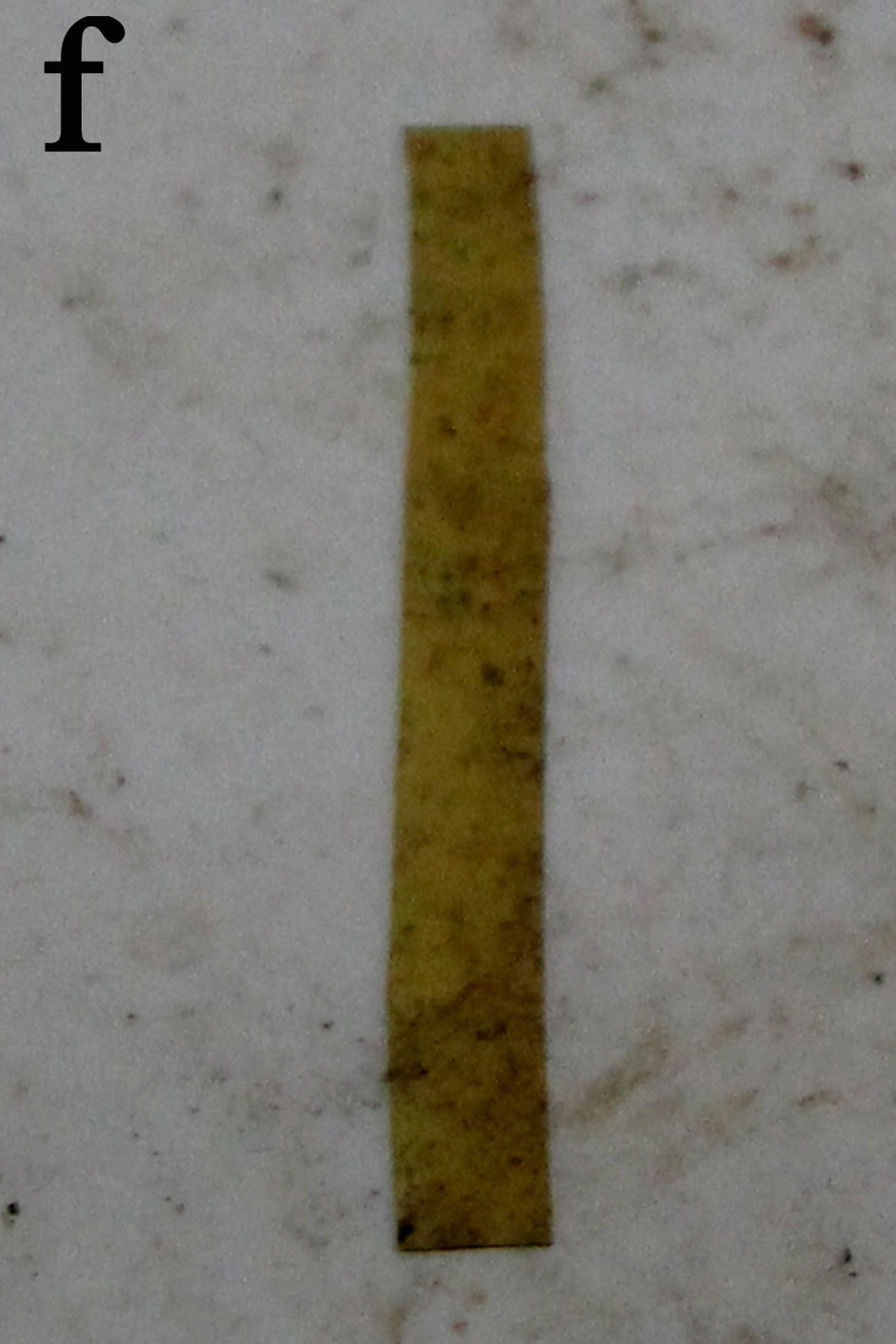

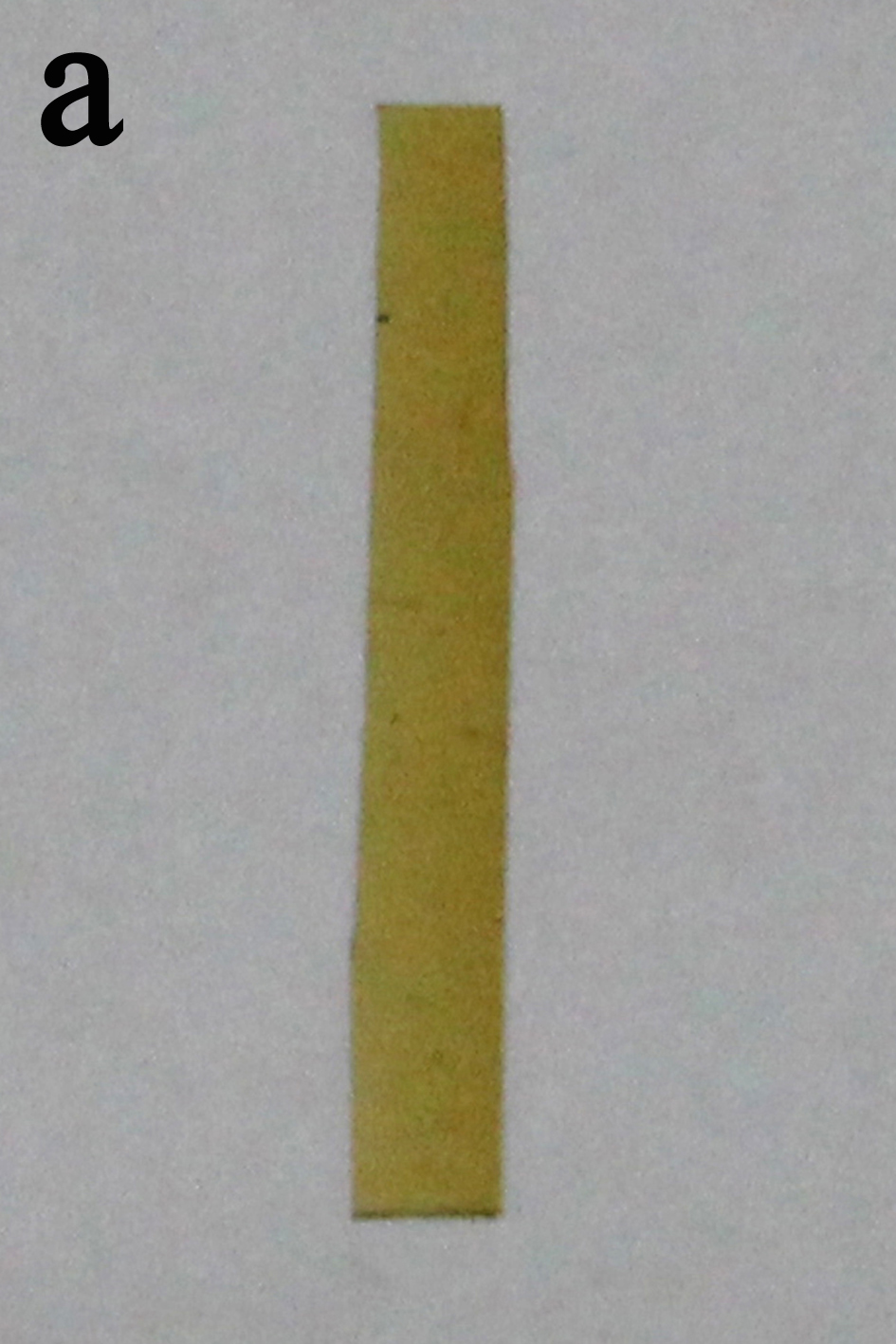

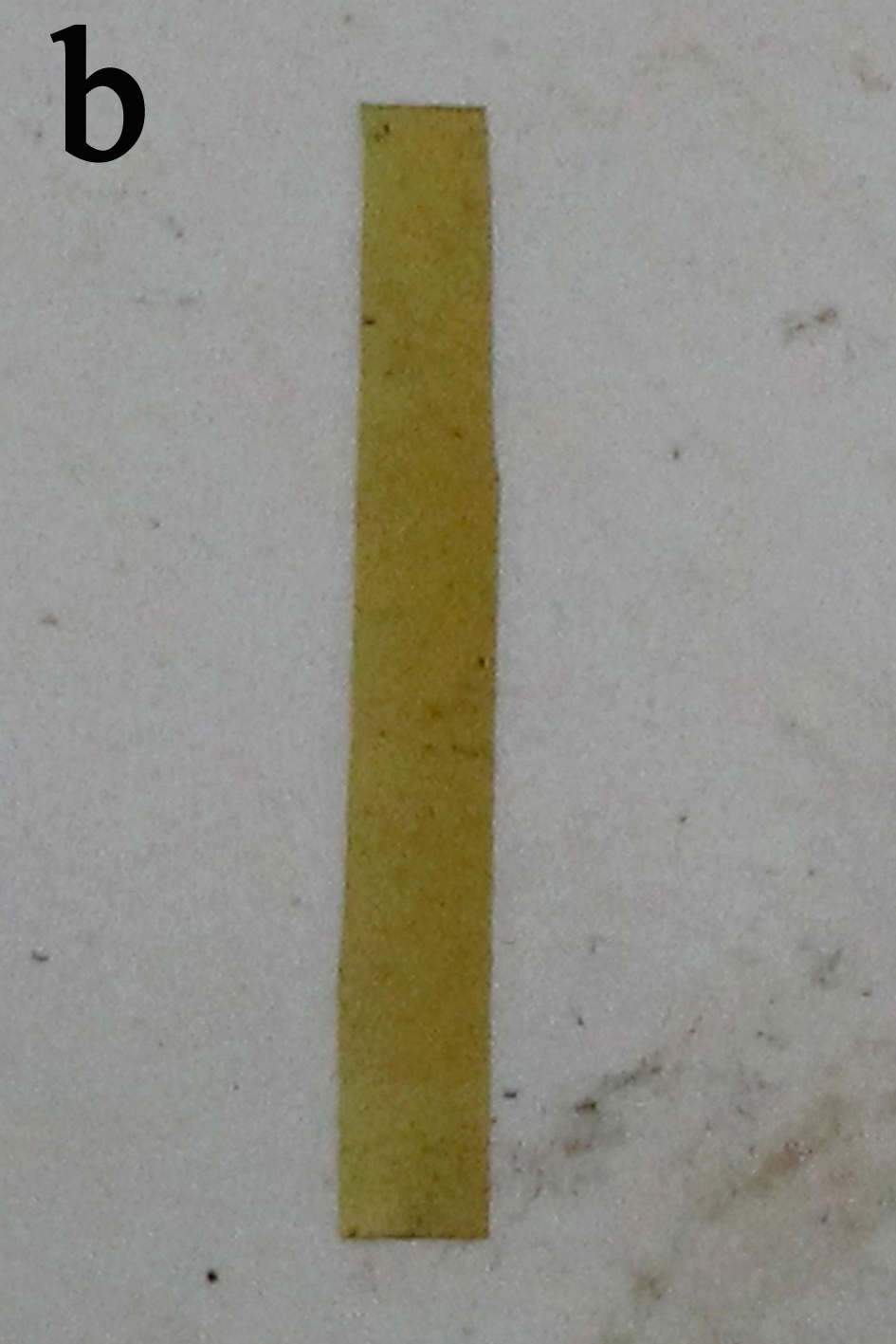

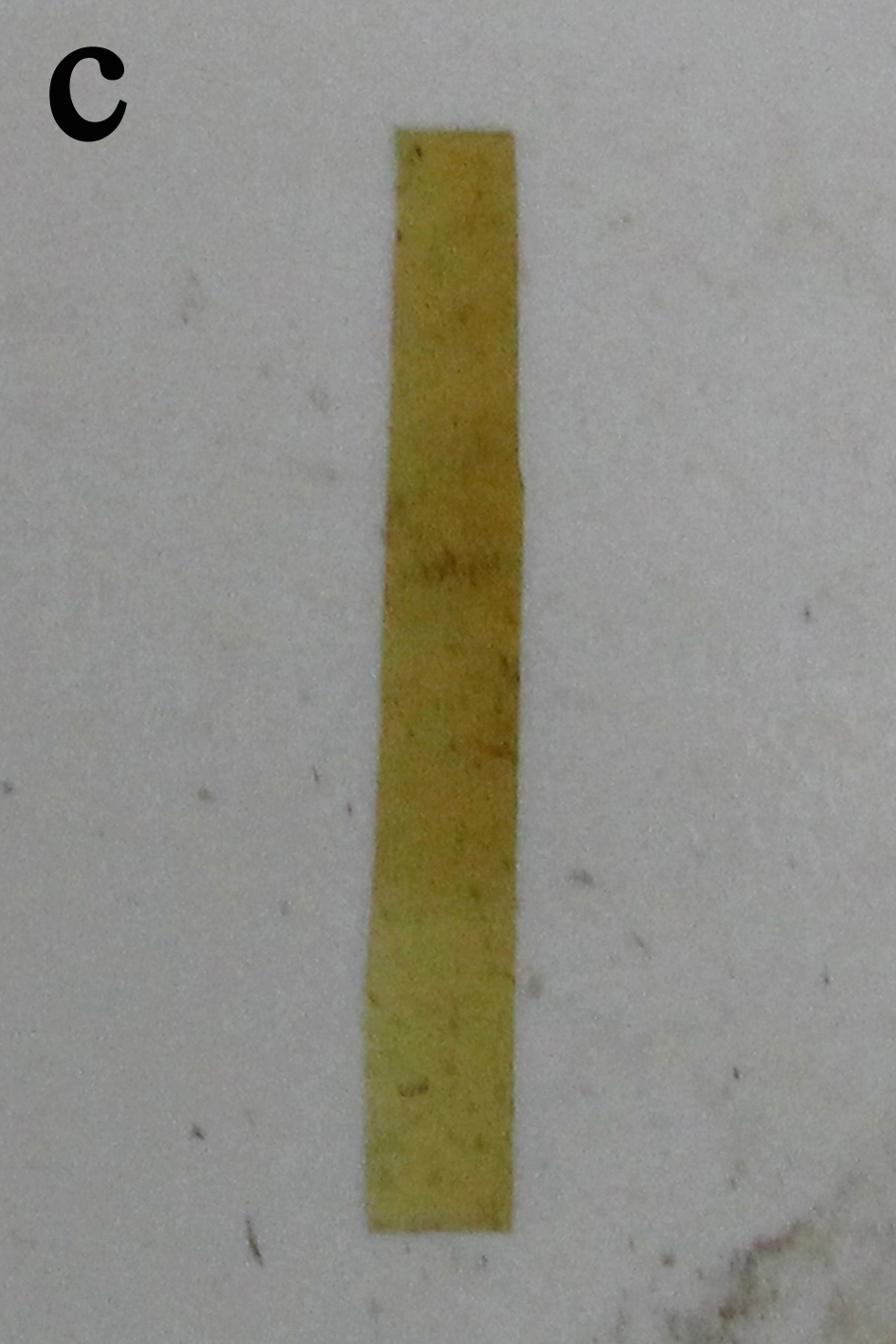


**Figure S3. Complete shape recovery of the high cycle-life shape memory polyimide at recovery temperatures from *T_g_*+20 °C to *T_g_*+70 °C.** (a) at *T_g_*+20 °C, (b) at *T_g_*+30 °C, (c) at *T_g_*+40 °C, (d) at *T_g_*+50 °C, (e) at *T_g_*+60 °C and (f) at *T_g_*+70 °C.


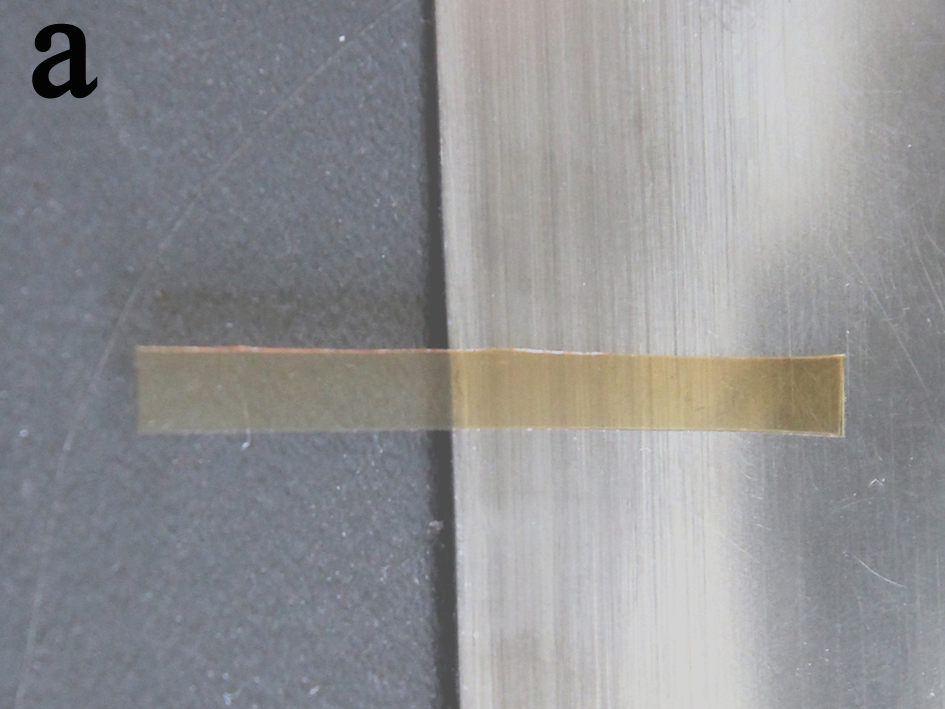

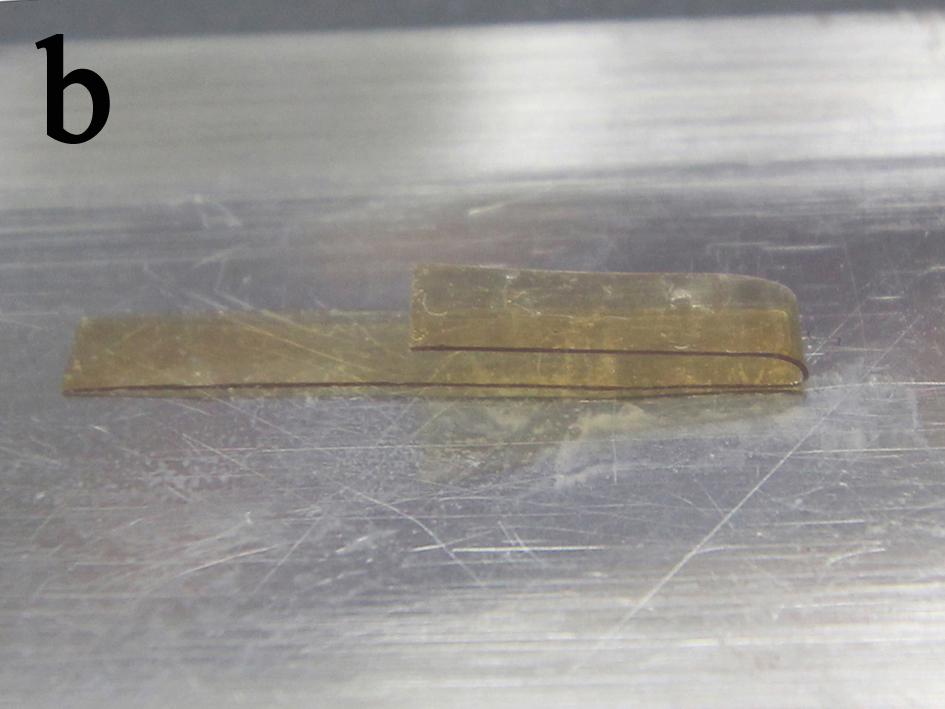

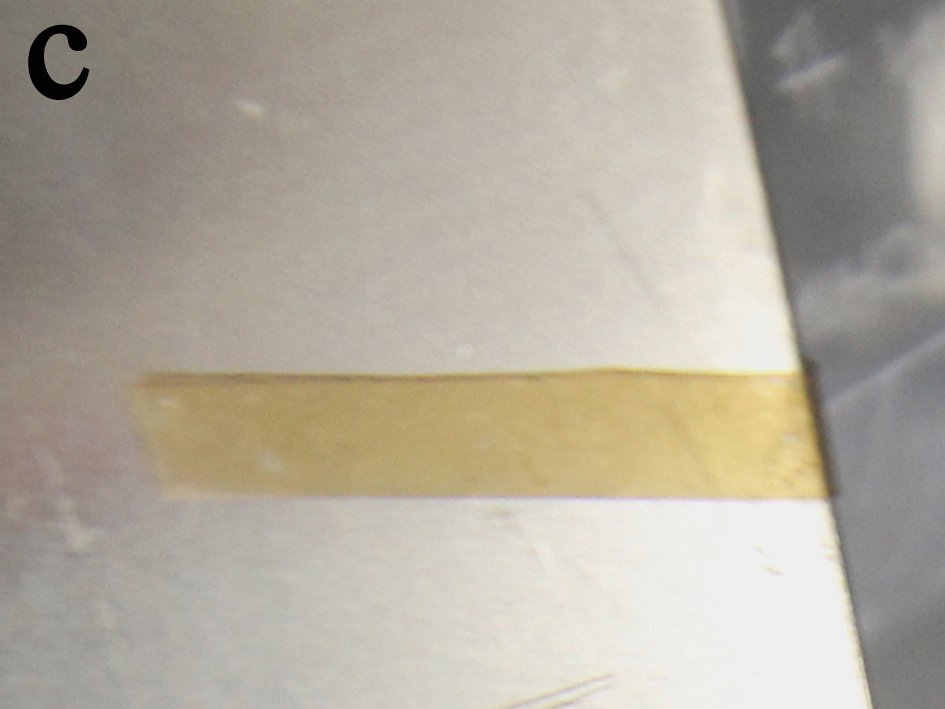

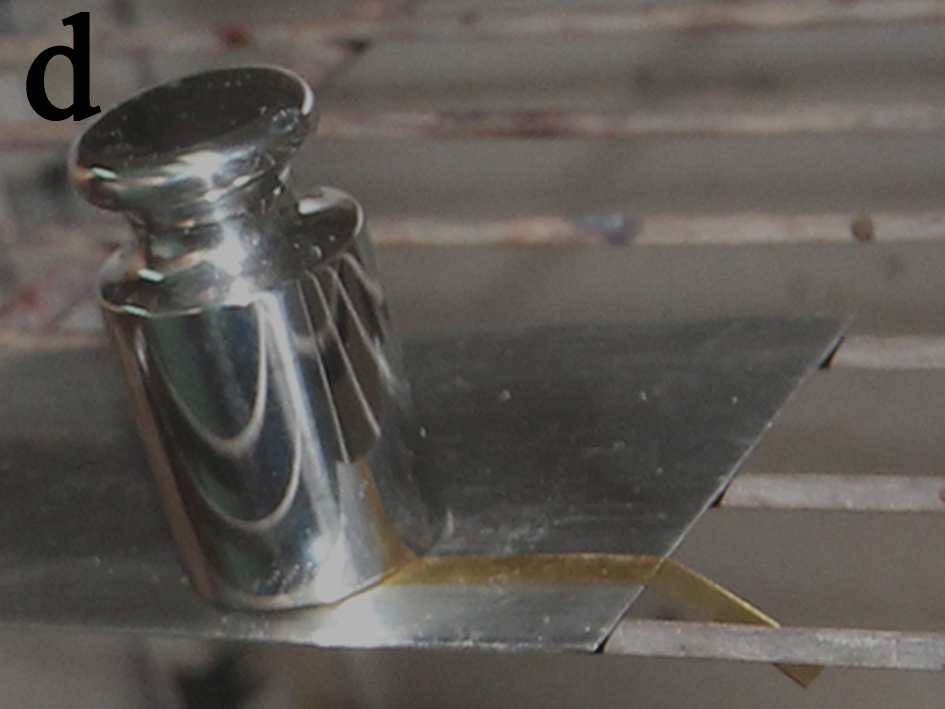

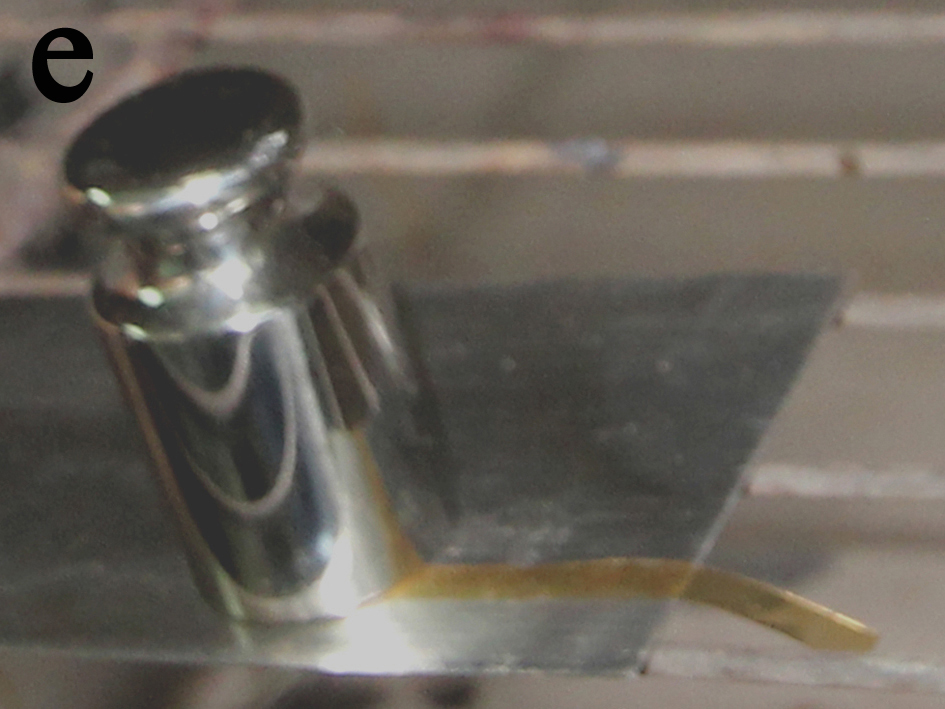

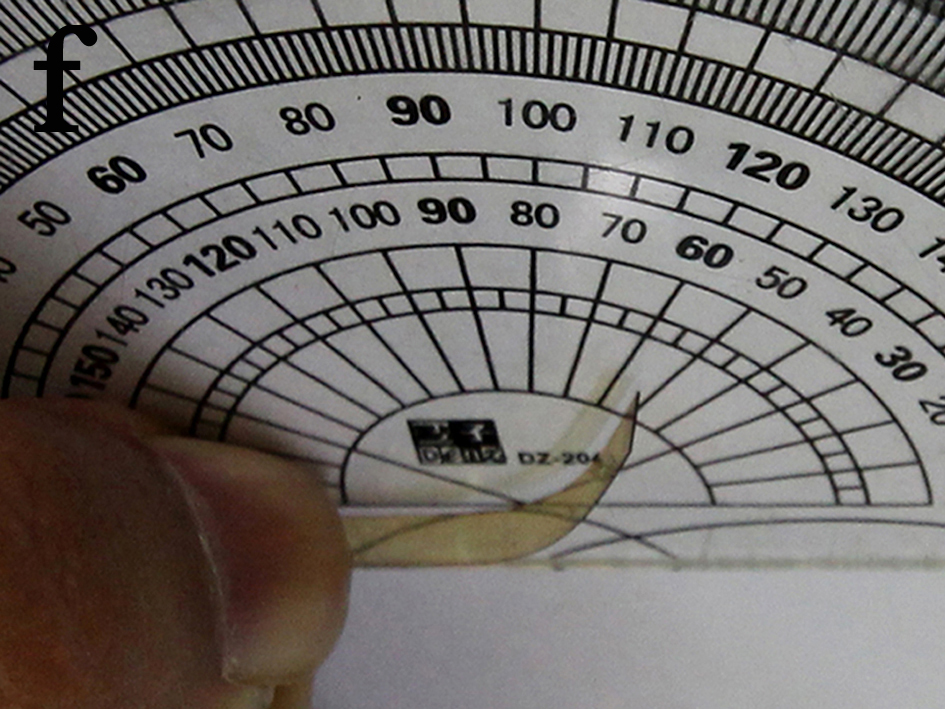


**Figure S4.** Shape recovery of the polyimide against gravitation. (a) initial flat lath, (b) bended temporary shape, (c) stainless-steel sheet placed inside the bended polyimide, (d) shape recovery against gravitation at *T_g_*, (e) shape recovery against gravitation at *T_g_*+20 °C and (f) illustration of recovery angle at *T_g_*+20 °C against gravitation.
